# The Coronavirus Network Explorer: mining a large-scale knowledge graph for effects of SARS-CoV-2 on host cell function

**DOI:** 10.1186/s12859-021-04148-x

**Published:** 2021-05-03

**Authors:** Andreas Krämer, Jean-Noël Billaud, Stuart Tugendreich, Dan Shiffman, Martin Jones, Jeff Green

**Affiliations:** Digital Insights, QIAGEN, Redwood City, USA

**Keywords:** COVID-19, Knowledge graph, Drug repurposing, Network biology

## Abstract

**Background:**

Leveraging previously identified viral interactions with human host proteins, we apply a machine learning-based approach to connect SARS-CoV-2 viral proteins to relevant host biological functions, diseases, and pathways in a large-scale knowledge graph derived from the biomedical literature. Our goal is to explore how SARS-CoV-2 could interfere with various host cell functions, and to identify drug targets amongst the host genes that could potentially be modulated against COVID-19 by repurposing existing drugs. The machine learning model employed here involves gene embeddings that leverage causal gene expression signatures curated from literature. In contrast to other network-based approaches for drug repurposing, our approach explicitly takes the direction of effects into account, distinguishing between activation and inhibition.

**Results:**

We have constructed 70 networks connecting SARS-CoV-2 viral proteins to various biological functions, diseases, and pathways reflecting viral biology, clinical observations, and co-morbidities in the context of COVID-19. Results are presented in the form of interactive network visualizations through a web interface, the Coronavirus Network Explorer (CNE), that allows exploration of underlying experimental evidence. We find that existing drugs targeting genes in those networks are strongly enriched in the set of drugs that are already in clinical trials against COVID-19.

**Conclusions:**

The approach presented here can identify biologically plausible hypotheses for COVID-19 pathogenesis, explicitly connected to the immunological, virological and pathological observations seen in SARS-CoV-2 infected patients. The discovery of repurposable drugs is driven by prior knowledge of relevant functional endpoints that reflect known viral biology or clinical observations, therefore suggesting potential mechanisms of action. We believe that the CNE offers relevant insights that go beyond more conventional network approaches, and can be a valuable tool for drug repurposing. The CNE is available at https://digitalinsights.qiagen.com/coronavirus-network-explorer.

**Supplementary Information:**

The online version contains supplementary material available at 10.1186/s12859-021-04148-x.

## Background

Severe acute respiratory syndrome coronavirus 2 (SARS-CoV-2), a member of the coronavirus family, is the etiologic agent of the pandemic COVID-19. Like other positive-stranded RNA viruses, its encoded proteins interact with proteins of the infected host cell at various stages of the replicative cycle, including with those involved in the immune response [1]. Such proteins therefore represent possible targets for the development of antiviral strategies. Human host proteins that bind to overexpressed SARS-CoV-2 viral proteins in immortalized human cells were previously identified using an affinity-purification mass spectrometry screen [2], which provides a starting point to study virus-host interactions using network-based approaches. Our goal in this paper is to illuminate possible molecular mechanisms permitting viral proteins to affect a range of host cell and immune functions that have been shown to be relevant in the context of COVID-19, and—in a second step—identify drugs that could potentially interfere with those mechanisms.

Host proteins that interact with viral proteins were initially functionally characterized and screened for existing drug targets against COVID-19 in [2]. Subsequent work expanded on this by further integrating SARS-CoV-2 viral proteins into the human interactome and using network biology approaches to identify existing drugs for repurposing [3–5], in some cases also leveraging gene expression data [1, 6]. Gysi et al. [5] provide a systematic exploration of state-of-the art proximity- and diffusion-based network algorithms, as well as algorithms based on graph convolutional networks with the primary goal of ranking candidate drugs for repurposing, but also exploring disease co-morbidities and tissue specificity using gene annotations.

In this work, we integrate SARS-CoV-2 viral proteins into a large-scale knowledge graph (KG) which in addition to including the protein interactome, also leverages various kinds of cause-effect relationships curated from the biomedical literature. In contrast to the approaches described above, these relationships specifically distinguish between activating and inhibiting effects therefore enabling predictions to be made about the direction of drug effects on host functions that are important in a clinical or disease context. For instance, clinical observations [7–11] indicate that SARS-CoV-2 has an activating effect on the coagulation of blood (severe coagulopathy has been detected in many patients with advanced disease) which leads us to specifically look for drugs that have an inhibiting effect on coagulation in order to counteract viral effects. The advantage of the method described here compared to the purely interactome-driven network biology approaches above is, that by integrating other experimental evidence from the literature in the form of cause-effect relationships, we are able to better elucidate relevant biological mechanisms, and propose repurposable drugs specifically targeted to block or counteract observed clinical endpoints.

Our algorithm uses a machine learning (ML) approach to prioritize genes that are known or predicted to causally affect a given host function either through activation or inhibition. This approach is based on the distributed representation [12] of genes as vectors embedded in a high-dimensional vector space. Such gene embeddings have been obtained previously from protein-protein interaction [13] and co-expression [14] networks, and were also used for function prediction [15, 16]. In contrast, here, we construct embeddings from known causal effects on the expression of other genes, curated from the literature, explicitly distinguishing between up- and down-regulation. This has the advantage that the direction of effects is already encoded in the embedding vectors.

In total, 70 networks involving viral proteins and a number of relevant “endpoint” functions were computed and made available for the community through a web interface called the Coronavirus Network Explorer (CNE). These networks represent the large spectrum of host biology affected by the viral infection. Immunological signaling pathways (e.g. IL-1, IL-6, IL-8) were included as they describe broadly the impact of the inflammatory setting in COVID-19 patients [17]. We have also included networks that display biological endpoints observed in severely or critically ill COVID-19 patients such as pneumonia, respiratory failure, and myocarditis [9, 10, 18, 19]. A set of networks represents the complex viral life cycle and its counterpart host response (e.g. replication, budding, entrance, antiviral response), and finally we included networks for functions that are possibly hijacked by the virus itself for its replication/multiplication or transmission (e.g. endocytosis and endoplasmic reticulum stress response) [20]. The complete list of included endpoint functions as they appear in the KG is shown in Additional file 1: Table S1.

## Implementation

### Overview of the algorithm

**Fig. 1:**
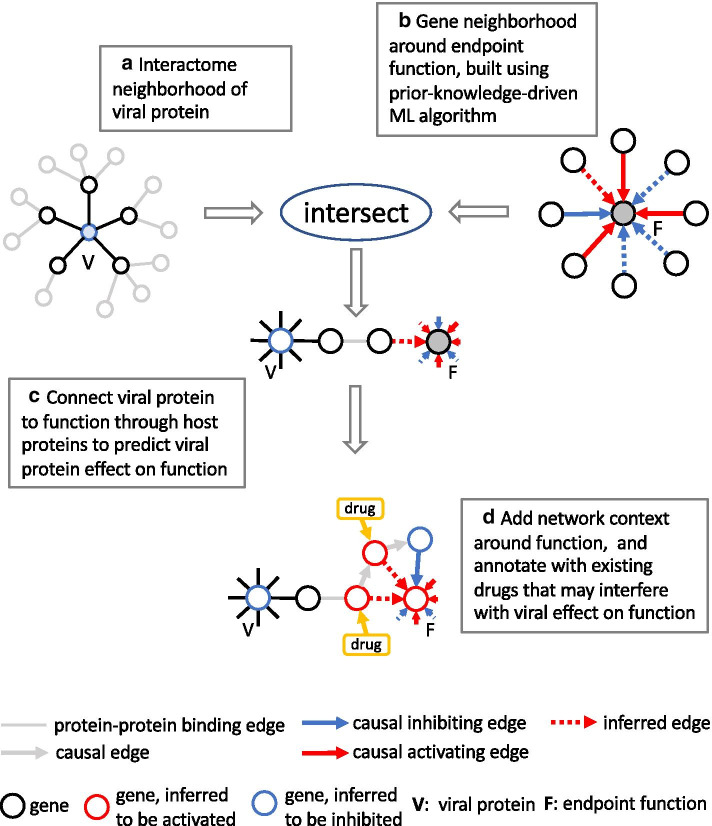
Method overview. **a** Network neighborhood around viral protein in the PPI, including direct interactors as well as specific second neighbors. **b** Gene neighborhood around endpoint function that contains genes that are prioritized or predicted to be important causal effectors based on an ML model leveraging the CGE and CGF. **c** Intersection of viral neighborhoods with function neighborhoods. **d** Addition of network context in the KG using a heuristic involving shortest paths. Genes in the resulting network are viewed as potential drug targets interfering with the functional effect of viral proteins, and are annotated with existing drugs

Three distinct subgraphs of the KG play specific roles in the different steps of our method: (1) the protein-protein interaction network (PPI), (2) the causal gene expression network (CGE), and (3) the causal gene-function network (CGF). The CGE contains relationships that describe the causal effect of genes on the expression of other genes, while the CGF is comprised of causal relationships between genes and functions, which can either be biological processes, diseases, or entire pathways. Figure 1 gives an overview of our approach: As a first step we build a network neighborhood around viral proteins in the PPI (Fig. 1a) that includes direct interactors as well as second neighbors that are “specific”, i.e. exclude network hubs. Using a ML algorithm that leverages the CGE and CGF, we then build gene neighborhoods around functions comprised of genes that are prioritized or predicted to be important causal effectors (Fig. 1b). Viral proteins are then connected to functions by intersecting both, viral and function neighborhoods (Fig. 1c). In order to create additional network context, subnetworks constructed in the previous step are then expanded by adding additional genes from the function neighborhood using a heuristic based on paths through the KG. Genes in the resulting network are viewed as potential drug targets interfering with the effect of viral proteins on the particular function, and are therefore annotated with existing drugs as candidates for repurposing (Fig. 1d).

### Knowledge graph

The KG is a large-scale network with approximately 120,000 nodes and 3.7 million edges, that represent prior knowledge from the biomedical literature. Nodes in the KG are genes, chemical compounds, drugs, microRNAs, and functions (biological processes, diseases, pathways). Different edge types represent a variety of functional mechanisms such as gene expression and transcription, activation and inhibition, phosphorylation, and protein-protein binding among others. The KG was constructed from the QIAGEN Knowledge Base [21, 22], a structured collection (using an ontology) of biomedical content that has been manually curated from the literature for the past 20 years, and also integrates content from third-party databases. The KG has three distinct subgraphs that play specific roles in the method presented here: the protein-protein interaction network (PPI), the causal gene expression network (CGE), and the causal gene-function network (CGF). The CGE contains relationships that describe the causal effect of genes on the expression of other genes, while the CGF is comprised of causal relationships between genes and functions. In addition, functions in the CGF are organized in a hierarchy (as part of the ontology) where generally parents inherit gene associations of their descendants. Each causal edge in the CGE and CGF has a positive or negative sign indicating the direction of the effect, i.e. whether it leads to an increase or decrease (or activation/inhibition). Most causal relationships represent experimental observations involving indirect effects. In the CGF, gene-pathway edges are treated in the same way as gene-function edges, i.e. genes are associated (through manual curation) with an activating or inhibiting effect on the pathway as a whole. All edges in the KG generally bundle a number of underlying literature findings from various experimental contexts, therefore edge signs reflect a consensus among all these contexts.

### Drugs and targets

Drug and target information was obtained from the QIAGEN Knowledge Base [22] which draws on other databases, e.g. DrugBank [23]. There are currently (as of December 19, 2020) 1533 drug targets and 4824 drugs (including combinations) represented in the QIAGEN Knowledge Base.

### Gene neighborhoods around viral proteins

We construct network neighborhoods around 27 viral proteins in the PPI that include direct interactors from Gordon et al. [2] as well as second neighbors (Fig. 1a). In total there are 330 host proteins directly binding to a viral protein, 41 of which can be mapped to the ML model. Second neighbor genes are added to this set provided that their interaction edge with a first neighbor is “specific”, i.e. the probability of finding such an edge in a random network with preserved node degrees is small. The idea behind this is that we want some confidence that the presence of a viral protein exerts an actual causal effect on the activity of genes in its neighborhood. This confidence is low for two-hop protein-protein interactions going through “hub” genes. In addition, including all second neighbors would lead to a combined viral neighborhood of 1453 genes out of 2314 (in the ML model), which is more that 63% of the complete network, so clearly unspecific. The probability of finding an edge between two given nodes $n_1$, $n_2$ in a random network preserving (expected) node degrees of the PPI is approximately $p=\frac{d_1d_2}{2E}$ (when $d_1$, $d_2$ are small enough) where $d_1$, $d_2$ are the degrees of nodes $n_1$, $n_2$, and *E* is the total number of network edges. We therefore construct a score $s = \left( \sum _{g'}\frac{2E}{d_{g'}d_g}\right) ^{-1}$ for each second neighbor gene *g* of a viral protein where the sum runs over all two-hop paths through the intermediate genes $g'$. The number of second neighbors included in the neighborhoods is then determined by a preselected score cut off $s_0$, and requiring $s<s_0$. For the CNE, we chose $s_0=0.001$ leading to a total size of the viral network neighborhood of 124 genes which is still roughly of the order of the number of direct interactors (and not of the size of the whole network). Increasing $s_0$ will make more genes available for intersection with function neighborhoods, but also introduce more noise (“false positives”), while decreasing $s_0$, possibly up to the point where no second neighbors are included, will increase the chance that important connections to functions are missed (“false negatives”). The results of this paper are not sensitive on the precise choice of $s_0$.

### Gene neighborhoods around functions

Gene neighborhoods around host functions are constructed from genes that are prioritized or predicted to be important causal effectors of a given function (Fig. 1b). For this we use a novel algorithm, based on machine learning, that leverages the CGE and CGF, and involves two parts, an unsupervised step to construct gene embeddings in a high-dimensional vector space, and a supervised step to score gene-function relationships. Building on an assumption that expression relationships encode information about gene function, the unsupervised step builds gene feature vectors by leveraging downstream causal gene expression signatures derived from the literature and represented in the CGE. It is important to distinguish this from gene expression patterns found in expression datasets: Here, expression signatures are created from individual published gene-to-gene expression or transcription relationships, not from datasets. The supervised step then employs a linear least-squares regression model using signed causal gene-function relationships in the CGF as training data. For the construction of gene neighborhoods around functions, the gene-function score is mapped to a *z*-score, and only genes that can be considered “significant” ($|z| > 2$) are included. Note, that the approach incorporates edge signs in the underlying networks. Therefore, unlike existing gene-function prediction approaches [24], it can distinguish between activating ($z>0$) and inhibiting ($z<0$) effects. High-scoring genes for a given function include both, genes that are already connected to the function by an edge in the CGF, as well as those that are purely predicted. These can be thought of exhibiting some “consistency” in the biology underlying the gene-function and gene-expression networks. Hence, the algorithm performs both, prediction and prioritization, since not all existing edges in the CGF necessarily have a high score.

#### Gene embedding

**Fig. 2:**
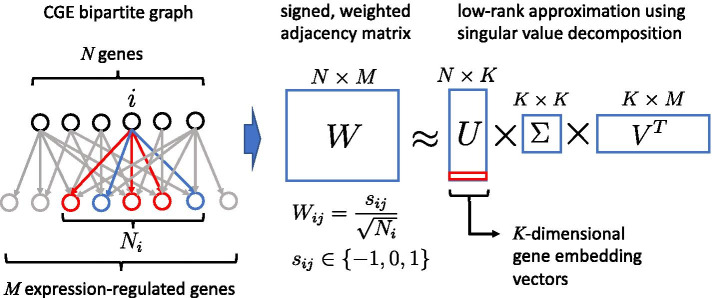
Gene embedding method based on the CGE bipartite graph with the signed, weighted adjacency matrix *W*. The rows of *W* represent regulating genes (for which embeddings are constructed), and columns are the genes of the downstream expression signature. The edge signs $s_{ij}$ are positive for upregulation, negative for downregulation, and $N_i$ is the total number of genes that are regulated by gene *i*. It is $s_{ij}=0$ if there is no edge. Gene embeddings are computed using a low-rank approximation of the singular value decomposition of *W*, $W\approx U \Sigma V^T$, where the matrix *U* projects one-hot encoded vectors representing single genes onto *K*-dimensional embedding vectors

In the following we view the CGE as a bipartite graph (see Fig. 2), i.e. regulating and regulated genes are distinguished from each other even if they refer to the same gene. We define the signed, weighted $N\times M$ adjacency matrix *W*, $W_{ij} = \frac{s_{ij}}{\sqrt{N_i}}$ with $s_{ij}\in \{-1, 0, 1\}$, whose rows represent the *N* genes for which we wish to compute embeddings, and columns are the genes of the downstream expression signature. The edge signs $s_{ij}$ are positive for upregulation, negative for downregulation, and $N_i$ is the total number of genes that are regulated by gene *i*. It is $s_{ij}=0$ if there is no edge. In order to compute gene embeddings we take the low-rank approximation using singular value decomposition [25] of *W*,

$$\begin{aligned} W\approx U \Sigma V^T, \end{aligned}$$

where columns of the $N\times K$ matrix *U* are the eigenvectors of the positive definite matrix $S=WW^T$, corresponding to its top *K* eigenvalues ($\Sigma$ is a diagonal $K\times K$ matrix and *V* is $M\times K$). We can think of *U* as projecting one-hot encoded vectors representing single genes onto *K*-dimensional embedding vectors, i.e. these embedding vectors are the rows of *U*. Note that $U^TU=I$. The weight factors $\frac{1}{\sqrt{N_i}}$ were chosen such that genes with different node degree in the CGE are put on equal footing. This is seen by noting that $S_{ii}=1$ for all *i*. For the CNE, the embedding dimension was set to $K=100$ (see Cross-validation). Also, in order to ensure that there is enough content coverage around the genes included in the model, we required included genes to have at least 10 downstream expression-regulated genes in the CGE.

#### Gene-function prediction and prioritization

Causal gene-function relationships from the CGF are captured in a signed bipartite adjacency matrix *Y*, with rows representing genes, and columns representing downstream functions. We only include those *N* genes that correspond to the rows of the matrix *W*. It is $Y_{ij} = 1$ if the effect of gene *i* exerted on function *j* is activating, and $Y_{ij} = -1$ if it is inhibiting, otherwise $Y_{ij} = 0$ if there is no edge between *i* and *j* in the CGF. The idea is to use the gene embedding vectors computed above as feature vectors in a linear model to predict the effect of gene *i* on function *j*. For each function *j* separately, we minimize the mean squared error

$$\begin{aligned} L_j = \frac{1}{N}\sum _{i}(\mathbf {x}_i\cdot \mathbf {\beta }_j-Y_{ij})^2 \end{aligned}$$

w.r.t. $\mathbf {\beta }_j$, where $\mathbf {x}_i$ is the *K*-dimensional gene embedding vector for gene *i*, and $\mathbf {\beta }_j$ is a *K*-dimensional parameter vector for function *j*. It follows that

$$\begin{aligned} \mathbf {\beta }_j = (U^TU)^{-1} U^T \mathbf {y}_j = U^T\mathbf {y}_j \end{aligned}$$

where $\mathbf {y}_j$ is the *j*th column vector of *Y*, and signed prediction “scores”

$$\begin{aligned} P_{ij} = \mathbf {x}_i\cdot \mathbf {\beta }_j \end{aligned}$$

are found to be orthogonal projections of $\mathbf {y}_j$ onto the subspace spanned by the column vectors of *U*,

$$\begin{aligned} P = UU^T Y. \end{aligned}$$

In order to make scores comparable across functions we map $P_{ij}$ to *z*-scores $Z_{ij}$ for each function *j* separately,

$$\begin{aligned} Z_{ij} = \frac{P_{ij}-\text{ mean}_i(P_{ij})}{\text{ std}_i(P_{ij})}, \end{aligned}$$

where mean and standard deviation are taken over all genes *i*. Like for the CGE, in order to ensure sufficient content coverage, we only considered functions that are connected to at least 10 genes in the CGF, i.e. have at least 10 non-zero entries in the matrix *Y*.

#### Cross-validation

Prediction accuracy of our model was tested using cross validation by randomly removing edges from the CGF, training the model, and then assessing how well those removed edges could be predicted. To avoid artificial dependencies between functions, we restricted ourselves to those that are “leaves” of the function hierarchy in the subset covered by the model. In order to create a balanced test set, we randomly picked *n* entries of the matrix *Y* that had the value 1, *n* entries that had the value -1, and 2*n* entries that were zero. This procedure was repeated *k* times to create *k* independent test sets. For each test set, the selected elements of *Y* were set to zero, a model was trained using this new matrix *Y*, and receiver-operating characteristic (ROC), and precision-recall curves were determined from the computed *z*-scores. It shall be noted that, strictly speaking, zero entries of *Y*, i.e. the lack of a gene-function edge in the CGF is not a true negative example in a training or test set since it only means that there is no finding in the literature regarding that causal gene-function effect. It does not mean that there was experimental evidence that this effect does not exist. We therefore make the (reasonable) assumption that the vast majority of zero-entries in *Y* correspond to true negative examples, and that the possible few “false” negative examples in the test set do not significantly affect test results. In that light, prediction can also be viewed as discovering the “false” negative entries in *Y*, i.e. missing edges in the CGF.

**Fig. 3:**
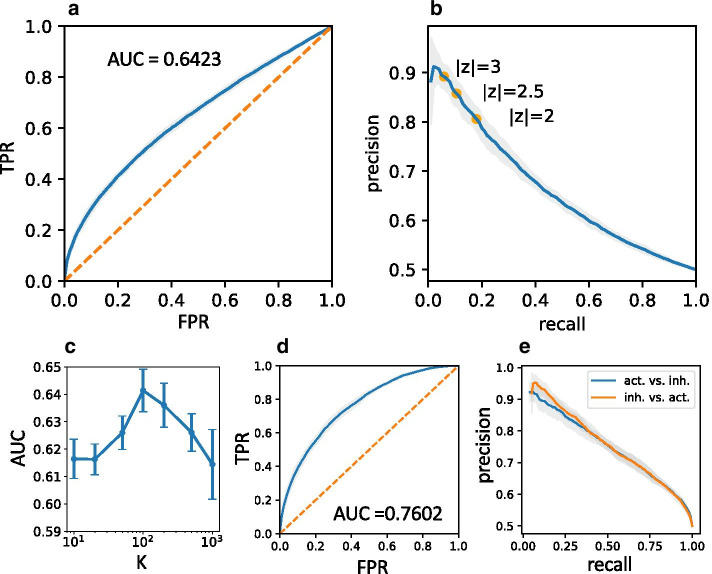
Cross validation of gene-function prediction. **a** Average ROC for edge prediction (TPR: true positive rate, FPR: false positive rate). **b** Average precision-recall curve for edge prediction indicating absolute z-score cut offs for different precision levels. **c** AUC for edge prediction as a function of the embedding dimenion *K*. The optimal embedding dimension is $K\approx 100$, smaller values of *K* lead to underfitting, while greater values lead to overfitting. **d** Average ROC for edge sign prediction. **e** Average precision-recall curves for edge sign prediction (blue: prediction of activation vs. inhibition, orange: prediction of inhibition vs. activation). Error bars and grey areas around curves represent standard deviations estimated from replicated tests

In particular, we assess two prediction tasks: (1) the prediction of the presence of an edge using a threshold on the absolute *z*-score, |*z*|, and (2) the prediction of the edge sign, i.e. whether its effect is activating or inhibiting, using thresholds on the *z*-score itself. For case (1) we use the complete test set with 4*n* examples, in case (2) the test set is limited to the 2*n* non-zero examples. Results are shown in Fig. 3 for $n=1000$, and $k=20$. Figure 3a, b show average ROC and precision-recall curves for case (1) for embedding dimension $K=100$, which is close to the optimal case w.r.t. the area under the ROC curve (AUC) (see Fig. 3c), where AUC $=0.6413$. For the construction of gene neighborhoods around functions we are interested in genes with the highest absolute *z*-scores ($|z|>2$ in the CNE) which corresponds to the case of low recall in our test scenario. As seen in Fig. 3b we reach about 90% precision in the limit of zero recall, while in the borderline case $|z|=2$ precision is around 80%. Case (2), which probes the discrimination between activating and inhibiting edges, has two sub-cases corresponding to the prediction of either activation or inhibition among edges with unknown sign, which leads to two separate precision-recall curves in Fig. 3e. We find a precision greater than 90% in the limit of zero recall for both sub-cases. Note, that only one ROC curve needs to be drawn for the first sub-case (see Fig. 3d), the ROC for the second sub-case is obtained by simply flipping the curve, leading to the same AUC $=0.7602$. As a cross check, to test whether there are no hidden biases, we have also run tests using random, normally distributed gene embedding vectors (i.e. random features that should not be reflective of any functional relationship), which, as expected, leads to an unpredictive model (AUC $\approx$ 0.5).

### Hypothesis network construction

Networks obtained by intersecting viral and function neighborhoods (see Fig. 1c) establish connections from a viral protein to a given endpoint function, possibly via a predicted gene-function relationship. Since, in general, these networks contain very few genes from the function neighborhood, we include additional genes with high absolute *z*-scores that can be connected through edges in the KG, and therefore likely play a role in the mechanism underlying the viral protein’s effect on the endpoint function. In order to do this, we employ a simple heuristic based on shortest paths (see Fig. 1d) through the KG from the viral protein to the endpoint function, where edge weights (i.e. single edge distances) are taken as the inverse geometric mean of the absolute *z*-scores of adjacent nodes (“*z*-scores” for nodes that don’t have one, e.g. the endpoint function, viral proteins, and some viral-interacting host proteins are set to 1). This choice leads to paths that are enriched in high-scoring genes from the function neighborhood. For the final hypothesis networks we take the union of all networks constructed from single viral proteins connecting to the same endpoint function. In some cases we have also included slightly longer paths to increase network size. Hypothesis networks are connected subgraphs of the KG that represent supporting experimental evidence from the literature for projected viral effects on the selected endpoint function.

### Software implementation and user interface

**Fig. 4:**
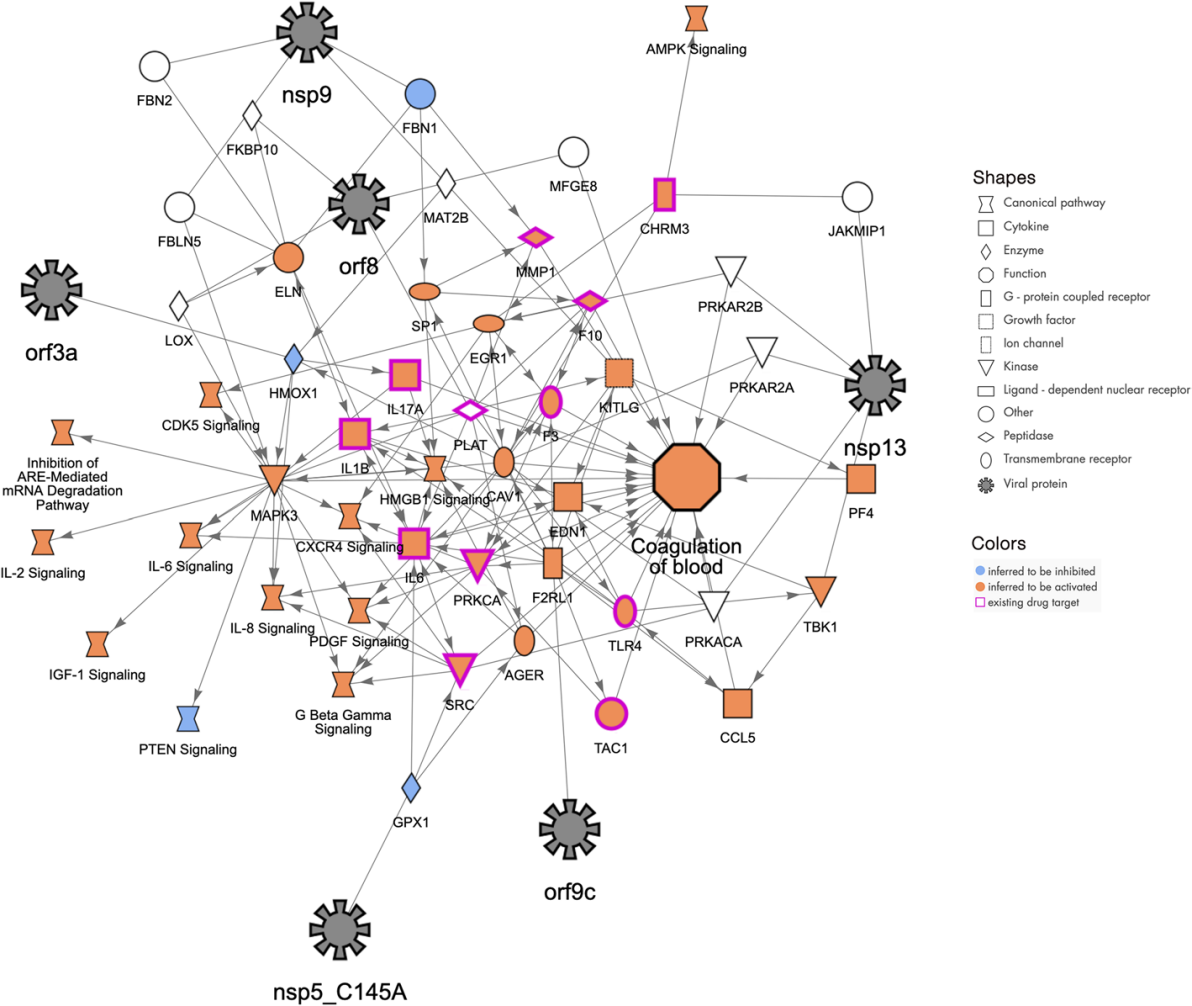
Molecular network that may explain how the host function “Coagulation of blood” can be increased by COVID-19 infection. The SARS-CoV-2 proteins (represented with black 8-pointed icons) bind to and may affect the activity of host proteins (various shapes) leading to increases in blood coagulation. The orange color of the host proteins and host function indicate predicted increases of activity whereas the blue color represents predicted decreases in activity. The network was constructed using the QIAGEN Knowledge Graph and machine learning techniques as described herein

**Fig. 5:**
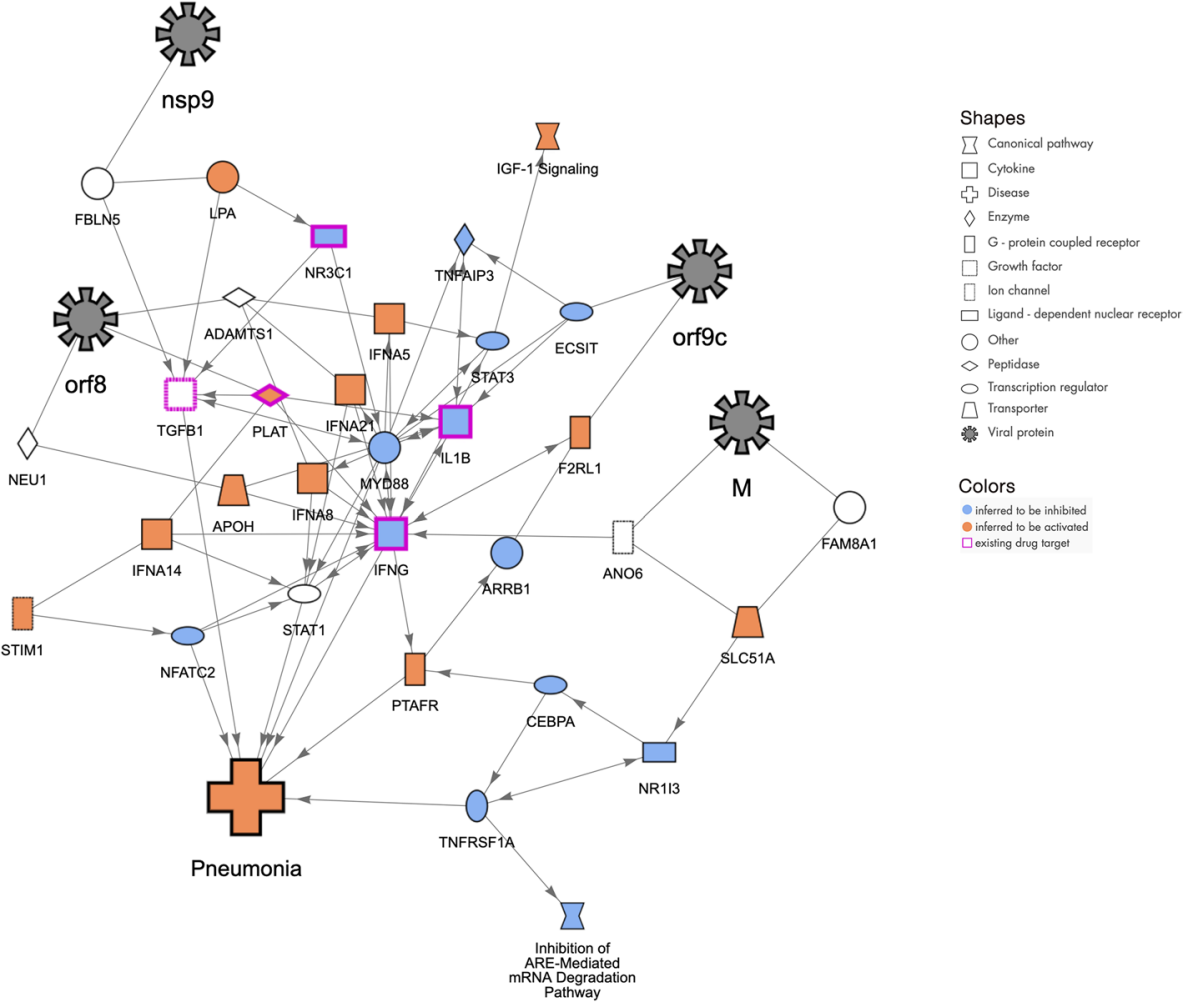
Molecular network that may explain how the host contracts “Pneumonia” by COVID-19 infection. The SARS-CoV-2 proteins (represented with black 8-pointed icons) bind to and may affect the activity of host proteins (various shapes) leading to increases in Pneumonia. The orange color of the host proteins and host function indicate predicted increases of activity whereas the blue color represents predicted decreases in activity. The network was constructed using the QIAGEN Knowledge Graph and machine learning techniques as described herein

**Fig. 6:**
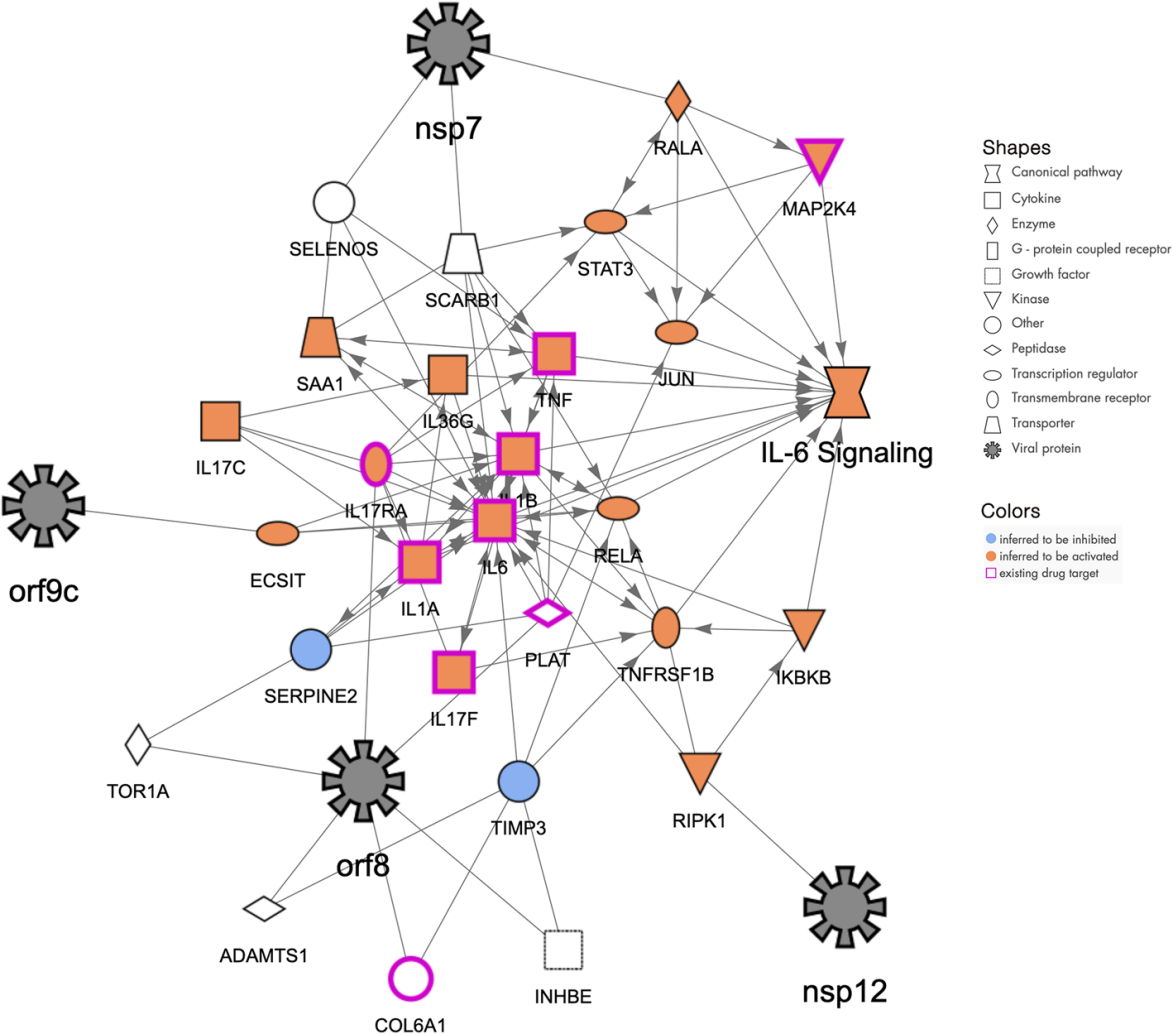
Molecular network that may explain how the host pathway “IL6 Signaling” can be increased by COVID-19 infection. The SARS-CoV-2 proteins (represented with black 8-pointed icons) bind to and may affect the activity of host proteins (various shapes) leading to increases in IL6 Signaling. The orange color of the host proteins and host function indicate predicted increases of activity whereas the blue color represents predicted decreases in activity. The network was constructed using the QIAGEN Knowledge Graph and machine learning techniques as described herein

The algorithm described here was implemented in Python using the standard scientific computing stack (numpy, pandas, sklearn, etc.). We also implemented a publicly accessible web-based interface through which the computed networks can be accessed for interactive exploration. This interface enables the user to select a particular endpoint function, as well as the direction of the effect (activating or inhibiting) that infection with SARS-CoV-2 is thought to have on this function. In the resulting displayed networks (see examples in Figs. 4, 5, 6) the user can click on any entity (node or edge) to reveal underlying findings from the literature, or a description of the selected entity itself. Colors of gene nodes indicate whether that particular gene is inferred to be activated (orange) or inhibited (blue) in order to achieve the preselected effect on the endpoint function. In addition, we also annotated genes with associated signaling pathways, colored according to their predicted activation state. Nodes whose activation status cannot be inferred, either because they are only connected by unsigned binding edges, or do not meet the score threshold, are shown in white. Genes that are targets for existing drugs are marked with a purple border. Selecting such a node will display the drugs targeting that gene along with each drug’s predicted effect on the endpoint function. The web application also allows users to search for specific genes or pathways in order to narrow down the number of networks to peruse.

## Results

### Functional analysis

The ML model (see Implementation) for constructing gene neighborhoods around functions (Fig. 1b) covers 2314 genes in total, all of which are also included in the PPI, and therefore potentially accessible in viral neighborhoods. Gene neighborhoods are computed for 11532 functions which include 276 pathways, 4155 diseases, and 7101 biological processes. The union of all first and second neighbors of SARS-CoV-2 viral proteins intersected with those covered by the ML model in total contains 124 genes. Function neighborhoods include on the average 108 genes (stdev: 18).

While the main objective of this work was to choose functional endpoints and construct corresponding networks using prior knowledge of COVID-19-related clinical observations and their underlying biology, we may also ask whether a statistical analysis of intersections of viral and function neighborhoods is able to pick up relevant biological processes, diseases, or pathways on its own without prior information. For this we computed Fisher’s Exact Test (FET) *p* values measuring enrichment of functional neighborhood genes in the set of 124 viral neighborhood genes for all 11532 functions. Significant functions ($p\le 0.05$) are shown in Additional file 1: Tables S2-S4. Notably we find fibrosis of heart ventricle, cardiotoxicity, and pulmonary alveolar proteinosis among the most significant diseases, and release of virus, the accumulation of triacylglycerol, dysfunction of endothelial tissue, and uptake of cholesterol among the most significant biological processes. This is not surprising, since pulmonary and cardiovascular complications are among the known manifestations of COVID-19. Also, since SARS-CoV-2 has a lipid envelope, cholesterol biosynthesis plays an important role in assembly and replication. Interestingly the list of significant functions also hints at neurological implications (e.g. injury of cerebrum, abnormal brain myelination, release of 5-hydroxytryptamine, exocytosis by neurons, and activation of dopaminergic neuron). Neurological effects are known to be observed in patients; for instance, there are widespread cases of loss of olfactory function and taste [26]. Some of the preselected functions that are included in the CNE also come up as significant: These are the fragmentation and degradation of Golgi apparatus, concentration of cholesterol, release of virus, CDK5 Signaling (which is involved in post-mitotic processes), and the diseases pneumonia, hypertension, and edema of pericardial cavity.

### Hypothesis networks

Hypothesis networks (Fig. 1d) were constructed for the 70 selected endpoint functions in Additional file 1: Table S1, and included in the CNE. Overall we find that 18 of the 27 SARS-CoV-2 viral proteins are represented in at least one of these networks, which contain on the average 13.6% (stdev: 5.3%) of the genes present in the neighborhood of a function (Fig. 1b). Examples of hypothesis networks are shown in Fig. 4 (coagulation of blood), Fig. 5 (pneumonia), and Fig. 6 (IL6 signaling). For the purpose of usability, in the CNE we have also annotated network nodes with relevant canonical pathways, provided gene and function descriptions, and show literature findings underlying edges. The CNE allows for an interactive exploration of gene and drug effects by preselecting the desired effect (promotion or suppression) on the endpoint function.

### Drugs and targets

We have assessed all hypothesis networks corresponding to the 70 endpoint functions in Additional file 1: Table S1 for included drugs and targets. We also specifically selected 24 of these endpoint functions (subset S) for which the direction of the desired drug effect (promotion or suppression) is evident (see Additional file 1: Table S1). For the following analysis, we only considered single-compound drugs, i.e. all drug combinations were excluded upfront. It shall also be noted that we only include drugs that have a host target that is included in the ML model. In total, we find 2382 drugs and 551 drug targets that are in principle accessible this way. There are 466 drugs and 118 corresponding targets that are present in at least one hypothesis network. Among them, 196 drugs are “consistent” (see Additional file 1: Table S5), i.e. for at least one of the 24 functions in the subset S, the computed drug effect on the function corresponds to the desired drug effect (promotion or suppression), 71 drugs are “inconsistent”, i.e. the opposite is the case, and for 199 drugs we could not easily determine the direction of the effect because information whether the drug acts as an agonist or antagonist was not available in the drug-target database (see Implementation) used, or the drug was not in any of the networks in subset S.

**Fig. 7:**
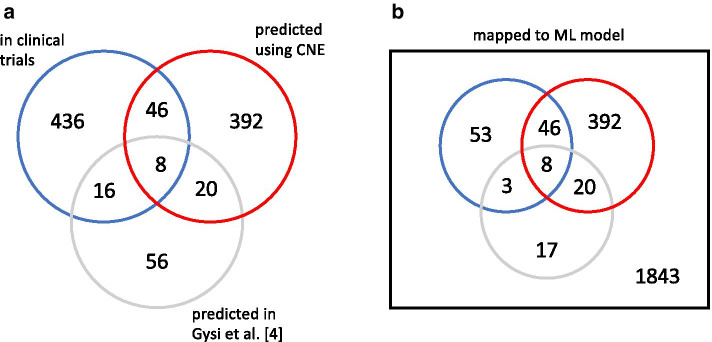
Venn diagram for drugs currently in clinical trials (from ClinicalTrials.gov [27]), included in CNE hypothesis networks, or predicted in [5]. **a** All drugs (excluding drug combinations). **b** Subset of drugs represented in our model. We find that 54 of the 466 drugs present in hypothesis networks are among the 110 drugs currently in clinical trials, and accessible in the ML model. Drugs present in hypothesis networks are strongly enriched in the set of clinical trial drugs (odds ratio: 4.35, FET *p* value: 8.10 $\times 10^{-13}$), and predicted drugs that are also in clinical trials, are enriched in the subset of drugs that are in multiple ($>5$) clinical trials (odds ratio: 4.69, FET *p* value: 0.00291). We compare our results to the set of 100 top-scoring drugs predicted by Gysi et al. [4] based on their consensus model, and added the corresponding numbers to the Venn diagrams. The number of clinical trial drugs among predicted drugs is 24 in their approach, while it is 54 in ours. 28 drugs predicted by Gysi et al. [4] are also found in hypothesis networks, and 8 of those drugs are at the same time present in the clinical trial set

In order to compare the 466 drugs found in our hypothesis networks to those currently in clinical trials, we downloaded a list of COVID-19 clinical trials from ClinicalTrials.gov [27], and after normalizing drug names and excluding drug combinations, assembled a table of 506 drugs, 110 of which could be mapped to the ML model. These drugs, together with the number of clinical trials in which they are currently tested, are listed in Additional file 1: Table S6, noting also which of them are predicted in a hypothesis network. As a result, we find that 54 of the 466 drugs present in hypothesis networks are among the 110 drugs currently in clinical trials, and accessible in the ML model. This is shown in the Venn diagrams in Fig. 7a (all drugs), and Fig. 7b (only drugs accessible in the ML model). As a further result we find that 30 of the overlapping drugs are consistent, 8 are inconsistent, and 16 have an unknown direction of effect. Drugs present in hypothesis networks are strongly enriched in the set of clinical trial drugs (odds ratio: 4.35, FET *p* value: 8.10 $\times 10^{-13}$), and predicted drugs that are also in clinical trials, are enriched in the subset of drugs that are in multiple ($>5$) clinical trials (odds ratio: 4.69, FET *p* value: 0.00291).

To make contact to other approaches that use network biology for predicting repurposable drugs, we compare our results to the set of 100 top-scoring drugs obtained by Gysi et al. [4] based on their consensus model, and added the corresponding numbers to the Venn diagrams in Fig. 7. The number of clinical trial drugs among predicted drugs is 24 in their approach, while it is 54 in ours. 28 drugs predicted by Gysi et al. [4] are also found in hypothesis networks, and 8 of those drugs are at the same time present in the clinical trial set. Interestingly, 6 of these drugs (dexamethasone, hydrocortisone, methylprednisolone, budesonide, prednisolone, and prednisone) have the same target, the glucocorticoid receptor (NR3C1), while the other two (secukinumab and rivaroxaban) target the proinflammatory cytokine IL17A, and the coagulation factor F10.

## Discussion

In contrast to existing network biology approaches [2–6] that are based on the interactome, the method presented here leverages literature-curated cause-effect relationships represented in the KG as additional information. It relies on an algorithm that prioritizes known causal gene-function relationships, and predicts new ones that are deemed most important for the underlying biology, including their direction of effect (activation or inhibition). This is used to construct “hypothesis networks”, subgraphs of the KG, that show related experimental observations from the literature, and therefore allow for biological interpretation to determine plausible molecular mechanisms in contrast to the more “black box”-like existing network approaches. We have applied our approach to drug repurposing by identifying existing targets affecting functional endpoints that either represent known viral effects, or clinical observations and co-morbidities. Drug predictions are therefore driven by external information in addition to their relationships to host proteins that interact with the virus. This has the advantage of providing better biological interpretability of predicted drug effects, but is in contrast to existing approaches [2–6] where no such information is used, and predictions are made solely based on network connectivity of drug targets. In addition, our approach has the ability to explicitly distinguish between activating and inhibiting drug effects which can be taken into account to select the most promising candidates for repurposing.

A comparison with the work of Gysi et al. [4] shows that their results for significant co-morbidities obtained by intersecting disease network modules with the COVID-19 module is in line with our findings from the functional analysis (see Results). In their list of 100 top-scoring drugs, 24 overlap with the list of drugs in clinical trials that we compiled (see Results). In contrast, 54 of the drugs covered by the 70 hypothesis networks that we constructed are also in the clinical trial list. An explanation could be that, since our method is driven by functional endpoints that reflect known clinical manifestations, it is closer to the medical community assessment regarding the efficacy of drugs which is reflected by the drugs currently in clinical trials. It shall be noted that the method presented here is limited to drugs that have a host target, so it will not be able to detect antiviral drugs that target viral proteins or RNA.

The CNE likely misses a number drugs (e.g. methotrexate and hydroxychloroquine are predicted in [4] but not in our approach) because their (relevant) target is currently not covered by the underlying ML model for gene-function prioritization which contains 2314 genes. This occurs because we took a conservative approach in requiring included genes to be sufficiently well-connected by signed edges in the KG (see Implementation). More genes could be added by either relaxing those requirements or curating additional content in the future. Similarly our approach is at this time limited by the use of the Gordon et al. data set [1] and could be expanded by including additional data. It shall be noted that our method does not involve any explicit scoring, and simply collects all drugs that modulate targets linked to viral-interacting host proteins and affect a given functional endpoint, so the number of drugs predicted depends on the set of endpoints included. Hypothesis networks expose experimental evidence from the literature as cause-effect relationships from the KG which helps elucidate underlying biological mechanisms. Ongoing work is focused on making those mechanisms more explicit, for instance by bringing in established causal cascades from known pathways, or adding tissue and cell type context.

In the following we discuss three hypothesis networks in more detail from a biological perspective.

### Coagulation of blood

Most people infected with SARS-CoV-2 will experience mild to moderate respiratory illness and recover promptly. However, in some cases, severe disease occurs with major pathophysiological and sometimes lethal outcomes. Comorbidities such as cardiovascular, renal, and respiratory preexisting conditions contribute to the severity seen in these patients. One drastic impact seen is the change in hemostasis after SARS-CoV-2 infection. Severe coagulopathy can arise and is associated with increased fatality rates in severely ill patients. The SARS-CoV-2 infection induces a pro-coagulative state and may result in vascular leakage and disseminated intravascular coagulation. The proinflammatory unbalance is thought to be one of the key factors of this uncontrolled clotting consequence seen in severe COVID-19 [7–11, 28]. The network presented here (Fig. 4) displays the interrelations between some of the key host molecules involved in the coagulation cascade, such as F3/tissue factor, F10, PLAT/plasminogen activator, and angiogenic factors or molecules related to angiogenesis balance such as F2RL1/proteinase-activated receptor 2 and EDN1/endothelin 1, PF4/Platelet factor 4 and several viral proteins (nsp9, nsp13, orf3a, orf9c, orf8).

The severity of COVID-19 is generally a consequence of hypercytokinemia (“cytokine storm”) with its dramatic increase of chemokines and their cellular consequences (e.g. increase of neutrophils, thrombocytopenia, endothelialitis). Therefore, this network also displays the contribution of increased pro-inflammatory molecules or signaling pathways (IL1B, IL6, IL8, IL17a) and upregulated chemokine signaling (CXCR4 signaling, CCL5) observed in severe COVID-19 outcomes. This network highlights the importance of understanding the molecular interplay between the players, and as shown recently, anticoagulant treatment appears to decrease mortality in severe COVID-19 patients.

### Pneumonia

Viral pneumonia with acute respiratory distress syndrome (ARDS) is one of the extreme consequences of COVID-19, a condition requiring mechanical ventilation as treatment. Patients with these severe conditions develop progressive respiratory failure following dramatic cascades of events. Dysfunctional immune responses in these patients will induce these events and are characterized by low IFN type I, III, a high pro-inflammatory setting, elevated chemokine secretion, high infiltration of myeloid and T cells in the lung, and finally severe pulmonary edema and pneumonia [29–31]. This network (Fig. 5) shows the possible interplay among type I Interferons, interleukins, the glucocorticoid receptor, sensors of viral infections and elements of the JAK/STAT pathway or the coagulation cascade and key coronaviral proteins that might promote pneumonia.

### IL6-Signaling

IL6 is an important pleiotropic interleukin that signals through the JAK/STAT (JAK1 and STAT3 in particular) and the MAPK pathway. It is expressed by immune cells (dendritic cells, macrophages, B cells and also epithelial cells) and it is involved in many biological processes including cell survival, apoptosis, maturation of T cells, TH1/Th2/Th17 differentiation/balance, and inflammation. IL6 is described as a pro-inflammatory cytokine, secreted in response to IL1B and TNF stimulation. The severe or critical cases of COVID-19 correlate with high levels of IL6 (during the cytokine storm) and low lymphocyte counts [18, 32–34]. Furthermore, higher levels of IL6 correlate with the risk of developing ARDS. As such, clinical trials are now ongoing and are designed to target IL6 using monoclonal antibody therapy (Tocilizumab and Sarilumab) as IL6 seems to be one of the key promoters of fatal outcomes. IL6 signaling described in this network (Fig. 6) includes all the major proinflammatory cytokines (IL1B, IL17, TNF and IL6,). Several key molecules of inflammation signaling are also present such as RELA, IKBKB, RIPK1 as well as MAPK signaling. It is thought that all these genes are either upregulated or predicted to be activated and would participate in the general increase of IL6 signaling and its unfortunate consequences in COVID-19.

## Conclusions

We have presented the Coronavirus Network Explorer (CNE), a tool to explore how SARS-CoV2 may interfere with various cell functions through interactions with host genes. Our approach involves “mining” of a large-scale literature-derived knowledge graph by connecting viral proteins to host genes that are predicted by a machine learning algorithm to be most relevant in a given functional context. The result is displayed as a network in which edges represent experimentally observed relationships between nodes, suggesting underlying molecular mechanisms of the pathogenesis of COVID-19. We have discussed a selection of these networks in order to demonstrate that our approach can identify biologically plausible hypotheses, grounded in actual immunological, virological and pathological observations seen in SARS-CoV-2 infected patients.

The CNE can also be used to find existing drugs that could be repurposed against COVID-19, as well as potentially novel drug targets. An important difference to other network-based approaches is that the discovery of repurposable drugs here is driven by prior knowledge of relevant functional endpoints that reflect known viral biology or clinical observations. Therefore, proposed drugs are supported by biological context suggesting potential mechanisms of action. Another advantage of our method is that it explicitly distinguishes between activating and inhibiting causal effects, so in the context of drug repurposing it can discriminate between agonists and antagonists. A survey of all drugs included in our networks finds that more than 50 of them are already in clinical trials, another indicator for the validity of our approach. Though there is still a number of improvements that can be made, we believe that the CNE offers relevant insights for COVID-19 pathogenesis that go beyond more conventional interactome-based network approaches, and can be a valuable tool for drug repurposing.

## Availability and requirements

- Project name: Coronavirus Network Explorer (CNE)
- Project home page: https://digitalinsights.qiagen.com/coronavirus-network-explorer
- Operating system(s): Platform-independent
- Programming language: N/A
- Other requirements: Modern web browser (Chrome, Firefox, or Safari)
- License: Networks created by the CNE are covered by the CC BY license.
- Restrictions to use by non-academics: None

## Supplementary Information

**Additional file 1.** Supplementary tables S1–S6.

**Additional file 2.** Jupyter notebook with example code and data.

## Data Availability

The Coronavirus Network Explorer (CNE) has been made freely available to the scientific community as a web-based resource at https://digitalinsights.qiagen.com/coronavirus-network-explorer. Each interactive network has an individual URL that can be shared independently. Networks can also be accessed computationally (code provided in a Jupyter notebook, see Additional  file 2), and are available within QIAGEN Ingenuity Pathway Analysis (IPA) (https://www.qiagen.com/qiagen-ipa) to allow exploration in the context of other datasets. IPA is commercially available. For reproducibility of the gene-function prediction method, example data and code are provided within a Jupyter notebook (see Additional  file 2).

## Abbreviations

CNE: Coronavirus Network Explorer
KG: knowledge graph
PPI: protein–protein interaction network
CGE: causal gene expression network
CGF: causal gene-function network
ML: machine learning
FET: Fisher’s Exact Test
ARDS: acute respiratory distress syndrome
ROC: receiver-operating characteristic
AUC: area under the ROC curve
