# Supplementary material for: The Coronavirus Network Explorer: mining a large-scale knowledge graph for effects of SARS-CoV-2 on host cell function
Source: BMC Bioinformatics. 2021 May 3;22:229. doi: 10.1186/s12859-021-04148-x (PMC8091149; doi:10.1186/s12859-021-04148-x)
Supplement: Supplementary file 1 — Additional file 1. Supplementary tables S1–S6. [file 12859_2021_4148_MOESM1_ESM.pdf]

Supplementary Tables

Supplementary Table S1: Selected endpoint functions. Those functions included in subset S have their respective sign (desired drug effect) in parentheses.

AMPK Signaling
Antiviral response (+1)
Apoptosis
Asthma
Autophagy
Budding of virus (-1)
CDK5 Signaling
CXCR4 Signaling
Cell cycle progression
Cellular homeostasis
Chemotaxis
Clathrin mediated endocytosis
Coagulation of blood (-1)
Concentration of cholesterol (-1)
Degradation of Golgi apparatus
Degranulation of cells
Diabetes mellitus
Edema of pericardial cavity
Endocytosis (-1)
Endoplasmic reticulum stress response of cells
Engulfment of cells
Entrance of virus (-1)
Exocytosis (-1)
Fibrosis (-1)
Fragmentation of Golgi apparatus
Fusion of cells
Fusion of phagosomes
Fusion of vesicles
G Beta Gamma Signaling
Glycolysis of cells
HIV infection (-1)
HMGB1 Signaling
Hemoglobinopathy
Hemostasis
Hypertension
I-kappaB kinase/NF-kappaB cascade
IGF-1 Signaling
IL-1 Signaling
IL-2 Signaling
IL-6 Signaling
IL-8 Signaling
Import of protein
Infection by RNA virus (-1)
Inflammation of respiratory system (-1)
Inhibition of ARE-Mediated mRNA Degradation Pathway
Internalization of protein
Interphase
M phase
Macroautophagy of cells
Macropinocytosis
Mitosis
Myocarditis (-1)
PDGF Signaling
PTEN Signaling
Phagocytosis
Pneumonia (-1)
Production of reactive oxygen species
Pulmonary Hypertension
Release of virus (-1)
Replication of RNA virus (-1)
Replication of coronavirus (-1)
Respiratory failure (-1)
SAPK/JNK Signaling
Stabilization of mRNA
Synthesis of lipid (-1)
Synthesis of phospholipid (-1)
Synthesis of reactive oxygen species
Transport of virus (-1)
Viral Infection
Viral life cycle (-1)

Supplementary Table S2: Significant biological processes.

	p-value	preselected endpoint
Accumulation of triacylglycerol	3.27341e-05	
Reproduction	5.83445e-05	
Release of 5-hydroxytryptamine	0.000156675	
Quantity of hdl cholesterol in blood	0.000516416	
Maturation of erythroid precursor cells	0.000519996	
Quantity of glycosphingolipid	0.000597035	
Esterification of cholesterol	0.00109144	
Quantity of sperm	0.0011722	
Incorporation of triacylglycerol	0.00130617	
Glucose tolerance	0.00177419	
Exocytosis by neurons	0.0022751	
Lean body mass	0.00256448	
Quantity of adipose tissue	0.00257632	
Excretion of protein	0.00267917	
Shape change of epithelial cell lines	0.00299916	
Formation of aggresome	0.00299916	
Uptake of cholesterol	0.00314775	
Quantity of chondrocytes	0.00321366	
Excitation of pyramidal neurons	0.00355631	
Vascular resistance of kidney	0.00368978	
Consumption of oxygen	0.0037325	
Quantity of burst-forming erythroid cells	0.0037325	
Fragmentation of Golgi apparatus	0.00386367	X
Quantity of palmitoleic acid	0.00386367	
Relaxation of cardiac muscle	0.00386367	
Degradation of Golgi apparatus	0.00386769	X
Synthesis of tretinoin	0.00419759	
Cell spreading of fibroblast cell lines	0.00500035	
Contraction of arteriole	0.00515229	
Relaxation of vein	0.0057544	
Activation of heart	0.0057544	
Focal necrosis	0.0057544	
Oncogene-induced senescence of cells	0.00579429	
Beta-oxidation of palmitic acid	0.006223	
Arousal	0.00632412	
Initiation of transcription	0.00666807	

Degranulation of natural killer cells	0.00669885	
Concentration of cholesterol	0.00717794	X
Cell viability of vascular smooth muscle cells	0.00744732	
Insulin sensitivity	0.00763836	
Release of beta-estradiol	0.00770903	
Release of virus	0.00775895	X
Percentage body fat	0.00820352	
Nitration of protein	0.00885116	
Development of erythroid cell lines	0.00895365	
Quantity of ldl cholesterol in blood	0.00937562	
Mass of organism	0.00937562	
M2 polarization	0.00937562	
Intravasation of cells	0.00959401	
Internalization by epithelial cell lines	0.00959401	
Development of respiratory tract	0.00961612	
Hydrolysis of cholesterol ester	0.00966051	
Contractility of left ventricle	0.00966051	
Erection	0.00990832	
Trafficking of leukocytes	0.0102802	
Secretion of 5-hydroxytryptamine	0.0102802	
Formation of dendritic spines	0.0103039	
Quantity of GH in blood	0.010666	
Transport of 5-hydroxytryptamine	0.0107895	
Cell proliferation of pituitary cancer cell lines	0.0107895	
Concentration of triacylglycerol	0.0108393	
Synthesis of triacylglycerol	0.0113501	
Inhibition of neurons	0.011995	
Formation of adrenal gland	0.011995	
Quantity of secretory vesicles	0.0131522	
Incorporation of long chain fatty acid	0.0133968	
Formation of lung	0.0137721	
Migration of kidney cells	0.0139316	
Secretion of granules	0.0139316	
Remodeling of artery	0.0142561	
Distribution of sphingolipid	0.0143549	
Secretion of epinephrine	0.0152055	
Concentration of D-glucose	0.0152757	
Energy expenditure	0.0163305	
Localization of phosphatidylserine	0.0169434	
Phagocytosis of T lymphocytes	0.0171791	

Proliferation of dermal fibroblasts	0.0171791	
Accumulation of hormone	0.0172187	
Beat of cardiomyocytes	0.0172982	
Extraintestinal functional disorder	0.0174582	
Efferocytosis	0.0179061	
Appetite	0.0179061	
Entrance of HIV-1	0.0184502	
Utilization of D-glucose	0.0186209	
Quantity of 5,6,7,8-tetrahydrobiopterin	0.0186209	
Angiogenesis of skin	0.0186209	
Quantity of long-chain acyl-coenzyme A	0.0190985	
Endurance	0.0193642	
Activation of dopaminergic neurons	0.0197242	
Exocytosis of synaptic vesicles	0.0199067	
Function of central nervous system	0.0200447	
Quantity of mitochondria	0.0203236	
Insulin sensitivity index	0.0204644	
Maturation of granulocytes	0.0210378	
Translocation of carbohydrate	0.0215278	
Concentration of bile acid	0.0215278	
Quantity of cerebral cortex cells	0.0215278	
Relaxation of aortic ring tissue	0.0215278	
Release of GABA	0.0215278	
Quantity of ketone body	0.0215774	
Quantity of retinal ganglion cells	0.021727	
Strength of muscle	0.021727	
Survival of red blood cells	0.0224905	
Proliferation of oligodendrocyte precursor cells	0.0228034	
Increased clearance of creatinine	0.0231739	
Release of norepinephrine	0.0231739	
Quantity of glomerular cells	0.0239332	
Development of endoderm	0.024322	
Macroautophagy	0.024322	
Migration of peripheral blood lymphocytes	0.024322	
Fractional shortening of left ventricle	0.024322	
Binding of E box motif	0.024322	
Adipogenesis of adipoblasts	0.024322	
Distribution of glycolipid	0.0246037	
Acoustic startle response	0.0247172	
Premature senescence of lung cell lines	0.0247172	

Basal metabolic rate	0.0253513	
Synthesis of cholesterol ester	0.025527	
Contraction of heart ventricle	0.0257632	
Homing of fibroblast cell lines	0.0258226	
Conversion of sterol	0.0264241	
Pressure of heart	0.0264241	
Survival of dopaminergic neurons	0.0264241	
Translocation of lipid	0.0264241	
Development of pulmonary artery	0.0271644	
Innervation of cells	0.0271644	
Concentration of tretinoin	0.0273527	
Apoptosis of B lymphoblastoid cell lines	0.0281838	
Cell viability of neuroglia	0.0281838	
Mobility of mice	0.0281838	
Quantity of TNF in blood	0.0287078	
Patterning of cells	0.0288403	
Hydroxylation of steroid	0.0288403	
Uptake of polyunsaturated fatty acids	0.0288403	
Quantity of endothelial cells	0.0289734	
Release of prostaglandin D2	0.0300608	
Apoptosis of effector T lymphocytes	0.0300608	
Movement of lipid	0.0300608	
Liquid preference	0.0302691	
Metabolism of sterol	0.0306196	
Formation of apoptotic bodies	0.0306196	
Vasodilation of artery	0.0309742	
Long-term memory	0.0311172	
Quantity of acetylcholine	0.0320627	
Migration of nervous tissue cell lines	0.0320627	
Interaction of bone marrow stromal cells	0.0325087	
Apoptosis of heart	0.0335738	
Efflux of choline-phospholipid	0.0341193	
Contraction of pulmonary artery	0.0341193	
Body temperature	0.0341979	
Deposition of glycogen	0.0341979	
Spermiogenesis	0.034435	
Quantity of IgG3	0.0360579	
Binding of cellular membrane	0.0363078	
Migration of neuroblasts	0.0363078	
Biosynthesis of stearic acid	0.0363078	

Cell division of lymphocytes	0.0363915	
Cytotoxic reaction of cells	0.0370681	
Mass of abdominal fat pad	0.0370681	
Maturation of megakaryocytes	0.0380189	
Senescence of dermal fibroblasts	0.0380189	
Adhesion of glomerular cells	0.0385478	
Induction of cellular protrusions	0.0385478	
Response of blood platelets	0.0385478	
Synthesis of norepinephrine	0.0385478	
Relaxation of coronary artery	0.0385478	
Place aversion	0.0385478	
Quantity of ovarian cortex cells	0.0389942	
Migration of lung cancer cell lines	0.0389942	
Quantity of islet cells	0.0389942	
Cell movement of cytotoxic T cells	0.0399945	
Deposition of triacylglycerol	0.0399945	
Regeneration of nerves	0.0403645	
Contraction of portal vein	0.0403645	
Cardiovascular toxicity	0.0405509	
Quantity of lysosome	0.040738	
Activation of peritoneal mast cells	0.040738	
Depression-related behavior	0.0408319	
Transmission of sterol	0.0408319	
Development of liver	0.0408319	
Modification of anion	0.0415911	
Conversion of hormone	0.0415911	
Metabolism of porphyrin	0.0418794	
Proliferation of Schwann cells	0.0420727	
Vertical rearing	0.0420727	
Metabolism of polyamines	0.0429536	
Habituation	0.0429536	
Oxidation of hormone	0.0429536	
Myelination of cells	0.0429536	
Cell spreading of kidney cell lines	0.0429536	
Oxidation of D-glucose	0.0429536	
Quantity of taurine	0.0429536	
Quantity of motor neurons	0.0433511	
Relaxation of corpus cavernosum penis	0.0433511	
Quantity of insulin in blood	0.0433511	
Oxidation of protein	0.0442588	

Flux of L-amino acid	0.0451856	
Export of metal ion	0.0457088	
Blood pressure	0.0457088	
Accumulation of lipid droplets	0.0457088	
Chemotaxis of tumor cells	0.0458142	
Cell viability of dermal cells	0.0458142	
Quantity of lysophosphatidylcholine	0.0458142	
Photoresponse	0.0477529	
Quantity of ganglion cells	0.0477529	
Cell death of megakaryocytes	0.0477529	
Ejection fraction of left ventricle	0.0485289	
Binding of vesicles	0.0485289	
Permeability of blood-brain barrier	0.0485289	
Synthesis of dihydrotestosterone	0.0485289	
Morphogenesis of head	0.0488652	

Supplementary Table S3: Significant diseases.

	p-value	preselected endpoint
Quantity of gastric ulcer	0.000461318	
Hypophagia	0.000620869	
Atrial fibrillation	0.00143219	
Musculoskeletal pain	0.00196336	
Allergic rhinitis	0.00267917	
Damage of retina	0.00326588	
Cholangitis	0.00334195	
Fibrosis of heart ventricle	0.00386367	
Dysfunction of endothelial tissue	0.00438531	
Cardiotoxicity	0.00453942	
Steatohepatitis	0.00454988	
Pulmonary alveolar proteinosis	0.00463447	
Foam cells	0.00480839	
Weight gain	0.00555904	
Abnormality of left ventricle	0.00744732	
Formation of gallstone	0.00744732	
Wheeze	0.00809096	
Diabetic nephropathy	0.00877001	
Injury of cerebrum	0.00879023	
Nonalcoholic fatty liver disease	0.0102802	
Fibrosis of interstitial tissue	0.011749	
Lysosomal storage disease	0.011995	
Duchenne muscular dystrophy	0.0127057	
Myocardial infarction	0.0127057	
Fibrosis of connective tissue	0.0128529	
Exencephaly	0.0128529	
Hip fracture	0.0143549	
Coronary artery disease	0.0149624	
Metabolic syndrome X	0.0156315	
Interstitial fibrosis	0.0157398	
Degeneration of outer nuclear layer	0.0160694	
Syndactyly	0.016788	
Valvular regurgitation of semilunar valve	0.0171791	
Atherogenesis	0.0186209	
Alcoholism	0.0190985	
Hyperplasia of apud cells	0.0204644	

Abnormality of atrium	0.0210378	
Hyperglycemia	0.021727	
Primary hypertension	0.0226464	
Sleep Disorders	0.0226464	
Fibrosis of muscle	0.0229615	
Hyperplasia of adrenal gland	0.0230675	
Damage of cardiac muscle	0.0230675	
Stenosis of vasculature	0.0231739	
Diastolic dysfunction	0.0231739	
Insulin tolerance	0.0240436	
Obesity	0.024322	
Fibrosis of renal glomerulus	0.0247172	
Synostosis	0.0258226	
Formation of ulcer	0.0264241	
Oxidative stress	0.0266686	
Inflammation of spinal cord	0.0271644	
Fatigue of muscle	0.0273527	
Sedation	0.0288403	
Atrophy of skin	0.0306196	
Pneumonia	0.0309415	X
Hypertension	0.0309415	X
Angina pectoris	0.0311172	
Bradycardia	0.0325087	
Impaired glucose tolerance	0.0325087	
Formation of granulation tissue	0.0341979	
Abnormal brain myelination	0.034435	
Damage of cerebral cortex	0.0353997	
Chronic kidney disease	0.0353997	
Severe arthritis	0.036141	
Dysfunction of heart	0.0363078	
Bleeding of skin	0.0363078	
Hypertrophic cardiomyopathy	0.0363078	
Thrombotic microangiopathy	0.0363078	
Ptosis	0.0380189	
Beta thalassemia	0.0387258	
Hypoalbuminemia	0.0415911	
Dysplasia of bone	0.0429536	
Dryness of skin	0.0429536	
Edema of pericardial cavity	0.0442588	X
Hypomyelination	0.0442588	

Non-insulin-dependent diabetes mellitus	0.0445656	
Akinesia	0.0445656	
Gliosis of central nervous system	0.0445656	
Postoperative nausea and vomiting	0.0451856	
Anovulation	0.0452898	
Immunosuppression	0.0457088	
Hypertriglyceridemia	0.0465586	
Huntington Disease	0.0476431	
Fibrosis of blood vessel	0.0477529	
Vascular injury	0.0477529	
Chronic brain damage	0.0485289	
Cirrhosis of liver	0.0485289	
Albuminuria	0.0485289	
Anophthalmia or microphthalmia	0.0488652	
Viral Infection	0.0540653	X

Supplementary Table S4: Significant pathways.

	p-value	preselected endpoint
Dopamine Receptor Signaling	0.00332482	
Signaling by Rho Family GTPases	0.0137595	
Sonic Hedgehog Signaling	0.0182462	
Paxillin Signaling	0.0217485	
Calcium Signaling	0.032472	
UVA-Induced MAPK Signaling	0.0379926	
Xenobiotic Metabolism PXR Signaling Pathway	0.0400179	
Cdc42 Signaling	0.0421185	
CDK5 Signaling	0.0453108	X
Apelin Adipocyte Signaling Pathway	0.0484869	
Coagulation System	0.0488837	

Supplementary Table S5: Consistent drugs included in hypothesis networks.

drug	targets	endpoint functions
6-aminocaproic acid	PLAT	pneumonia
ABT-414	EGFR	entrance of virus
AC0010MA	EGFR	entrance of virus
AP32788	EGFR	entrance of virus
AZD3759	EGFR	entrance of virus
BPI-7711	EGFR	entrance of virus
D-0316	EGFR	entrance of virus
FG-3019	CCN2	fibrosis
GS-5745	MMP9	hiv infection, replication of coronavirus, viral infection
HS-10296	EGFR	entrance of virus
KX-01	SRC	coagulation of blood, entrance of virus, exocytosis
N-methylscopolamine	CHRM3	coagulation of blood, exocytosis, hiv infection, synthesis of phospholipid
PEG-interferon alfa-2a	IFNAR1	release of virus, replication of coronavirus, viral infection, viral life cycle
abciximab	ITGAV, ITGB3	entrance of virus, fibrosis
acitretin	RXRB	inflammation of respiratory system
aclidinium	CHRM3	coagulation of blood, exocytosis, hiv infection, synthesis of phospholipid
adapalene	RXRB	inflammation of respiratory system
afatinib	EGFR	entrance of virus
aflibercept	VEGFA	entrance of virus, synthesis of phospholipid
alcaftadine	HRH1	inflammation of respiratory system, release of virus, viral life cycle
alflutinib	EGFR	entrance of virus
alirocumab	PCSK9	concentration of cholesterol
alitretinoin	RXRB	inflammation of respiratory system
amiloride	SLC9A1	budding of virus
anakinra	IL1R1	fibrosis
antazoline	HRH1	inflammation of respiratory system, release of virus, viral life cycle
apixaban	F10	coagulation of blood
argatroban	F2R	exocytosis, synthesis of phospholipid
aripiprazole	HRH1	inflammation of respiratory system, release of virus, viral life cycle
asenapine	HRH1	inflammation of respiratory system, release of virus, viral life cycle

atracurium	CHRNA3	pulmonary hypertension
atropine	CHRM3	coagulation of blood, exocytosis, hiv infection, synthesis of phospholipid
azelastine	HRH1	inflammation of respiratory system, release of virus, viral life cycle
becaplermin	PDGFRB	respiratory failure
beclomethasone	NR3C1	pneumonia
beclomethasone dipropionate	NR3C1	pneumonia
benztropine	CHRM3	coagulation of blood, exocytosis, hiv infection, synthesis of phospholipid
bepotastine	HRH1	inflammation of respiratory system, release of virus, viral life cycle
betamethasone	NR3C1	pneumonia
betamethasone dipropionate	NR3C1	pneumonia
betamethasone valerate	NR3C1	pneumonia
betrixaban	F10	coagulation of blood
bexarotene	RXRB	inflammation of respiratory system
bilastine	HRH1	inflammation of respiratory system, release of virus, viral life cycle
binimetinib	MAPK1	replication of rna virus, viral infection
biperiden	CHRM3	coagulation of blood, exocytosis, hiv infection, synthesis of phospholipid
bivalirudin	F2R	exocytosis, synthesis of phospholipid
bosutinib	SRC	coagulation of blood, entrance of virus, exocytosis
brigatinib	EGFR	entrance of virus
brompheniramine	CHRM3, HRH1	coagulation of blood, exocytosis, hiv infection, inflammation of respiratory system, release of virus, synthesis of phospholipid, viral life cycle
capivasertib	AKT1	exocytosis, hiv infection, infection by rna virus
carbinoxamine	CHRM3, HRH1	coagulation of blood, exocytosis, hiv infection, inflammation of respiratory system, release of virus, synthesis of phospholipid, viral life cycle
cariprazine	HRH1	inflammation of respiratory system, release of virus, viral life cycle
cetirizine	HRH1	inflammation of respiratory system, release of virus, viral life cycle
cetuximab	EGFR	entrance of virus
chloropromazine	HRH1	inflammation of respiratory system, release of virus, viral life cycle
ciclesonide	NR3C1	pneumonia
cilengitide	ITGAV, ITGB3	entrance of virus, fibrosis

cisatracurium	CHRM3, CHRNA3	coagulation of blood, exocytosis, hiv infection, pulmonary hypertension, synthesis of phospholipid
clemastine	HRH1	inflammation of respiratory system, release of virus, viral life cycle
clobetasol propionate	NR3C1	pneumonia
clocortolone pivalate	NR3C1	pneumonia
cortisone acetate	NR3C1	pneumonia
cyproheptadine	HRH1	inflammation of respiratory system, release of virus, viral life cycle
dacomitinib	EGFR	entrance of virus
dalteparin	F10, VEGFA	coagulation of blood, entrance of virus, synthesis of phospholipid
darifenacin	CHRM3	coagulation of blood, exocytosis, hiv infection, synthesis of phospholipid
dasatinib	SRC	coagulation of blood, entrance of virus, exocytosis
daunorubicin	POR	clathrin mediated endocytosis, concentration of cholesterol, entrance of virus
deacylcortivazol	NR3C1	pneumonia
deligoparin	F10	coagulation of blood
desloratadine	HRH1	inflammation of respiratory system, release of virus, viral life cycle
desonide	NR3C1	pneumonia
desoximetasone	NR3C1	pneumonia
dexamethasone	NR3C1	pneumonia
dexamethasone 21-acetate	NR3C1	pneumonia
dexamethasone phosphate	NR3C1	pneumonia
dexchlorpheniramine	HRH1	inflammation of respiratory system, release of virus, viral life cycle
difluprednate	NR3C1	pneumonia
diphenhydramine	CHRM3, HRH1	coagulation of blood, exocytosis, hiv infection, inflammation of respiratory system, release of virus, synthesis of phospholipid, viral life cycle
doxepin	CHRM3, HRH1	coagulation of blood, exocytosis, hiv infection, inflammation of respiratory system, release of virus, synthesis of phospholipid, viral life cycle
doxorubicin	POR	clathrin mediated endocytosis, concentration of cholesterol, entrance of virus
doxylamine	HRH1	inflammation of respiratory system, release of virus, viral life cycle
edoxaban	F10	coagulation of blood
emedastine	HRH1	inflammation of respiratory system, release of virus, viral life cycle
enoxaparin	F10	coagulation of blood

enzastaurin	AKT1, AKT2	exocytosis, hiv infection, infection by rna virus, pulmonary hypertension
epinastine	HRH1	inflammation of respiratory system, release of virus, viral life cycle
eptifibatide	ITGB3	entrance of virus
eritoran	TLR4	coagulation of blood
erlotinib	EGFR	entrance of virus
etanercept	TNF	transport of virus
etretinate	RXRB	inflammation of respiratory system
ezetimibe	NPC1L1, SOAT1	concentration of cholesterol
fesoterodine	CHRM3	coagulation of blood, exocytosis, hiv infection, synthesis of phospholipid
fevipiprant	PTGDR2	pulmonary hypertension
fexofenadine	HRH1	inflammation of respiratory system, release of virus, viral life cycle
flavoxate	CHRM3	coagulation of blood, exocytosis, hiv infection, synthesis of phospholipid
flunisolide	NR3C1	pneumonia
fluocinolone acetonide	NR3C1	pneumonia
fluocinonide	NR3C1	pneumonia
fluorometholone	NR3C1	pneumonia
flurandrenolide	NR3C1	pneumonia
fluticasone	NR3C1	pneumonia
fluticasone furoate	NR3C1	pneumonia
fluticasone propionate	NR3C1	pneumonia
fondaparinux	F10	coagulation of blood
gallium nitrate	IL1B	coagulation of blood, endocytosis, synthesis of phospholipid
gefitinib	EGFR	entrance of virus
glycopyrrolate	CHRM3	coagulation of blood, exocytosis, hiv infection, synthesis of phospholipid
halcinonide	NR3C1	pneumonia
halobetasol propionate	NR3C1	pneumonia
hydrocortisone buteprate	NR3C1	pneumonia
hydrocortisone butyrate	NR3C1	pneumonia
hydrocortisone cypionate	NR3C1	pneumonia
hydrocortisone succinate	NR3C1	pneumonia
hydroxyzine	HRH1	inflammation of respiratory system, release of virus, viral life cycle
hyoscyamine	CHRM3	coagulation of blood, exocytosis, hiv infection, synthesis of phospholipid

icotinib	EGFR	entrance of virus
idraparinux	F10	coagulation of blood
idronoxil	XIAP	viral infection
indomethacin	PLA2G2A	synthesis of lipid
infliximab	TNF	transport of virus
interferon alfacon-1	IFNAR1	release of virus, replication of coronavirus, viral infection, viral life cycle
interferon beta-1a	IFNAR1	release of virus, replication of coronavirus, viral infection, viral life cycle
interferon beta-1b	IFNAR1	release of virus, replication of coronavirus, viral infection, viral life cycle
ipatasertib	AKT1	exocytosis, hiv infection, infection by rna virus
ipratropium	CHRM3	coagulation of blood, exocytosis, hiv infection, synthesis of phospholipid
isoflurane	CHRNA3	pulmonary hypertension
ixekizumab	IL17A	coagulation of blood
ketotifen	HRH1	inflammation of respiratory system, release of virus, viral life cycle
lapaquistat	FDFT1	concentration of cholesterol
lapatinib	EGFR	entrance of virus
lazertinib	EGFR	entrance of virus
levamisole	CHRNA3	release of virus, replication of coronavirus
levocabastine	HRH1	inflammation of respiratory system, release of virus, viral life cycle
levocetirizine	HRH1	inflammation of respiratory system, release of virus, viral life cycle
loratadine	HRH1	inflammation of respiratory system, release of virus, viral life cycle
loteprednol etabonate	NR3C1	pneumonia
loxapine	HRH1	inflammation of respiratory system, release of virus, viral life cycle
marimastat	MMP1, MMP9	coagulation of blood, hiv infection, replication of coronavirus, viral infection
mecamylamine	CHRNA3	pulmonary hypertension
meclizine	HRH1	inflammation of respiratory system, release of virus, viral life cycle
mepenzolic acid	CHRM3	coagulation of blood, exocytosis, hiv infection, synthesis of phospholipid
methylprednisolone	NR3C1	pneumonia
methylprednisolone acetate	NR3C1	pneumonia
methylprednisolone succinate	NR3C1	pneumonia

mirtazapine	HRH1	inflammation of respiratory system, release of virus, viral life cycle
mitapivat	EIF2AK2	viral life cycle
naquotinib	EGFR	entrance of virus
nazartinib	EGFR	entrance of virus
neratinib	EGFR	entrance of virus
nimotuzumab	EGFR	entrance of virus
nintedanib	SRC	coagulation of blood, entrance of virus, exocytosis
nitazoxanide	POR	clathrin mediated endocytosis, concentration of cholesterol, entrance of virus
olanzapine	CHRM3, HRH1	coagulation of blood, exocytosis, hiv infection, inflammation of respiratory system, release of virus, synthesis of phospholipid, viral life cycle
olopatadine	HRH1	inflammation of respiratory system, release of virus, viral life cycle
opipramol	HRH1	inflammation of respiratory system, release of virus, viral life cycle
orphenadrine	CHRM3, HRH1	coagulation of blood, exocytosis, hiv infection, inflammation of respiratory system, release of virus, synthesis of phospholipid, viral life cycle
osimertinib	EGFR	entrance of virus
oxybutynin	CHRM3	coagulation of blood, exocytosis, hiv infection, synthesis of phospholipid
pactimibe	SOAT1	concentration of cholesterol
paliperidone	HRH1	inflammation of respiratory system, release of virus, viral life cycle
paliperidone palmitate	HRH1	inflammation of respiratory system, release of virus, viral life cycle
pegaptanib	VEGFA	entrance of virus, synthesis of phospholipid
pegcetacoplan	C3	hiv infection
pegintron	IFNAR1	release of virus, replication of coronavirus, viral infection, viral life cycle
perifosine	AKT1	exocytosis, hiv infection, infection by rna virus
pheniramine	HRH1	inflammation of respiratory system, release of virus, viral life cycle
pomalidomide	TNF	transport of virus
prednisolone	NR3C1	pneumonia
prednisolone acetate	NR3C1	pneumonia
prednisolone phosphate	NR3C1	pneumonia
prednisone	NR3C1	pneumonia
prinomastat	MMP9	hiv infection, replication of coronavirus, viral infection
probucol	ABCA1	concentration of cholesterol
promethazine	HRH1	inflammation of respiratory system, release of virus, viral life cycle

propantheline	CHRM3	coagulation of blood, exocytosis, hiv infection, synthesis of phospholipid
pyrotinib	EGFR	entrance of virus
quetiapine	HRH1	inflammation of respiratory system, release of virus, viral life cycle
quinidine	CHRM3	coagulation of blood, exocytosis, hiv infection, synthesis of phospholipid
ranibizumab	VEGFA	entrance of virus, synthesis of phospholipid
rasagiline	BCL2	release of virus
rebimastat	MMP1, MMP9	coagulation of blood, hiv infection, replication of coronavirus, viral infection
recombinant interferon	IFNAR1	release of virus, replication of coronavirus, viral infection, viral life cycle
resatorvid	TLR4	coagulation of blood
revefenacin	CHRM3	coagulation of blood, exocytosis, hiv infection, synthesis of phospholipid
rimexolone	NR3C1	pneumonia
risperidone	HRH1	inflammation of respiratory system, release of virus, viral life cycle
rivaroxaban	F10	coagulation of blood
rociletinib	EGFR	entrance of virus
rocuronium	CHRNA3	pulmonary hypertension
rolapitant	TAC1	coagulation of blood, inflammation of respiratory system
saracatinib	SRC	coagulation of blood, entrance of virus, exocytosis
scopolamine	CHRM3	coagulation of blood, exocytosis, hiv infection, synthesis of phospholipid
secukinumab	IL17A	coagulation of blood
selonsertib	MAP3K5	fibrosis, pulmonary hypertension
siltuximab	IL6	coagulation of blood, endocytosis, transport of virus
solifenacin	CHRM3	coagulation of blood, exocytosis, hiv infection, synthesis of phospholipid
succinylcholine	CHRNA3	pulmonary hypertension
sucralfate	FGF2	respiratory failure
tazarotene	RXRB	inflammation of respiratory system
tesevatinib	EGFR	entrance of virus
tesmilifene	HRH1	inflammation of respiratory system, release of virus, viral life cycle
thalidomide	TNF	transport of virus
tifacogin	F10	coagulation of blood
tiotropium	CHRM3	coagulation of blood, exocytosis, hiv infection, synthesis of phospholipid
tirofiban	ITGB3	entrance of virus
tolterodine	CHRM3	coagulation of blood, exocytosis, hiv infection, synthesis of phospholipid

triamcinolone	NR3C1	pneumonia
triamcinolone acetonide	NR3C1	pneumonia
tropicamide	CHRM3	coagulation of blood, exocytosis, hiv infection, synthesis of phospholipid
trospium	CHRM3	coagulation of blood, exocytosis, hiv infection, synthesis of phospholipid
umeclidinium	CHRM3	coagulation of blood, exocytosis, hiv infection, synthesis of phospholipid
vandetanib	EGFR	entrance of virus
varespladib methyl	PLA2G2A, PLA2G5	synthesis of lipid
varlitinib	EGFR	entrance of virus
vecuronium	CHRNA3	pulmonary hypertension
vorapaxar	F2R	exocytosis, synthesis of phospholipid
ziprasidone	HRH1	inflammation of respiratory system, release of virus, viral life cycle
zoledronic acid	FDFT1	concentration of cholesterol

Supplementary Table S6: Drugs in clinical trials that are also included in the ML model.

Drug	Number of clinical trials	predicted	consistent	inconsistent
Hydroxychloroquine	191			
Tocilizumab	43	X		
Enoxaparin	25	X	X	
Dexamethasone	21	X	X	
Methylprednisolone	20	X	X	
Nitazoxanide	19	X	X	
Anakinra	17	X	X	
Ruxolitinib	16	X		
Baricitinib	12	X		
Interferonbeta-1a	12	X	X	
Sarilumab	10			
Aspirin	10	X		X
Losartan	10			
Rivaroxaban	9	X	X	
Clazakizumab	8	X		
Acalabrutinib	8			
Isotretinoin	7			
Prednisone	6	X	X	
Telmisartan	6	X		
Canakinumab	6	X		
Interferonbeta-1b	6	X	X	
Hydrocortisone	6	X	X	
Famotidine	5			
Tofacitinib	5	X		
Mavrilimumab	5			
Opaganib	4			
Sargramostim	4			
Apixaban	4	X	X	
Budesonide	4	X		X
Ciclesonide	4	X	X	
Maraviroc	4	X		X
Ibrutinib	3			
Naltrexone	3	X		
Bevacizumab	3	X		
Bicalutamide	3			
Sirolimus	3			

Dipyridamole	3			
Nintedanib	3	X	X	
Pioglitazone	3	X		
Imatinib	3	X		X
Fostamatinib	3			
Nivolumab	3			
Linagliptin	3			
Prednisolone	3	X	X	
Nicotine	3	X		
Abatacept	2			
Crizanlizumab	2			
Disulfiram	2	X		X
Duvelisib	2			
Ebselen	2	X		
Eculizumab	2			
Fluoxetine	2			
Fluvoxamine	2			
Infliximab	2	X	X	
Pentoxifylline	2			
Ravulizumab	2			
Siltuximab	2	X	X	
Tranexamicacid	2			
Enzalutamide	2			
Sildenafil	2			
Thalidomide	2	X	X	
Fondaparinux	2	X	X	
Ibuprofen	2	X		X
Iloprost	2			
Naproxen	1	X		X
Ambrisentan	1			
Amiodarone	1			
Aprepitant	1			
Aprotinin	1			
Argatroban	1	X	X	
Bardoxolone	1			
Bivalirudin	1	X	X	
Candesartan	1			
Captopril	1			
Decitabine	1	X		
Diphenhydramine	1	X	X	

Dutasteride	1			
Edoxaban	1	X	X	
Eritoran	1	X	X	
Estradiol	1			
Icatibant	1			
Indomethacin	1	X	X	
Isoflurane	1	X	X	
Ketotifen	1	X	X	
Lenalidomide	1			
Levamisole	1	X	X	
Masitinib	1			
Mefloquine	1			
Mometasone	1	X		X
Ozanimod	1			
Pacritinib	1	X		
Prazosin	1			
Proxalutamide	1			
Quetiapine	1	X	X	
Ramipril	1			
Regadenoson	1			
Rintatolimod	1	X		
Secukinumab	1	X	X	
Semaglutide	1			
Senicapoc	1			
Sitagliptin	1			
Spironolactone	1			
Tacrolimus	1			
Tenecteplase	1			
Tirofiban	1	X	X	
Toremifene	1			
Tramadol	1			
Ulinastatin	1	X		
Valsartan	1			
Zanubrutinib	1			
